# Profiles of Occupational Burnout in the Group of Representatives of High-Risk Professions in Poland

**DOI:** 10.3390/ijerph19106297

**Published:** 2022-05-22

**Authors:** Marta Makara-Studzińska, Agnieszka Kruczek, Agata Borzyszkowska, Maciej Załuski, Katarzyna Adamczyk, Małgorzata Anna Basińska

**Affiliations:** 1 Department of Health Psychology, Faculty of Health Science, Collegium Medicum, Jagiellonian University, 31-008 Cracow, Poland; marta.makara-studzinska@uj.edu.pl (M.M.-S.); maciej.zaluski@uj.edu.pl (M.Z.); katarzyna.1adamczyk@uj.edu.pl (K.A.); 2 Department of Clinical Psychology, Faculty of Psychology, Kazimierz Wielki University, 85-867 Bydgoszcz, Poland; agata.borzyszkowska@ukw.edu.pl (A.B.); basinska@ukw.edu.pl (M.A.B.)

**Keywords:** occupational burnout, high-risk professions, traffic controllers, firefighters, ECDs

## Abstract

(1) Background: Working in a high-risk profession is associated with taking on a large responsibility and risking loss of health or life. These professions include, among others, air traffic controllers, firefighters, and ECDs. People working in these professions are particularly vulnerable to experiencing high levels of stress and developing professional burnout syndrome. The aim of the conducted research was to assess the external and internal differences in the intensity of occupational burnout dimensions among representatives of high-risk occupations and to distinguish burnout profiles among them. (2) Methods: The total number of participants working in high–risk occupations who took part in the study was N = 1239, including the following job positions: air traffic controllers (*n* = 107), firefighters (*n* = 580), and ECDs (*n* = 558). The respondents completed the following self-report questionnaires: a structured survey and the Link Burnout Questionnaire. The following statistical tests were performed: cluster analysis, analysis of variance, and chi-square test of independence. (3) Results: The highest intensity of burnout dimensions was presented by representatives of ECDs. Profiles reflecting the types of occupational burnout were distinguished. The representatives of air traffic controllers demonstrated the following profiles: 1—low risk of burnout with a component of psychophysical exhaustion; 2—exhausted with a moderate tendency to disappointment; 3—burned out, ineffective, and uninvolved. The profiles of the ECDs were: 4—engaged, with a reduced sense of effectiveness; 5—with a reduced sense of effectiveness; 6—burned out with a low sense of effectiveness. However, the profiles of the firefighters were: 7—not burned out; 8—at risk of burnout; 9—exhausted with a tendency towards disappointment. Individuals representing the various burnout profiles differed in terms of the severity of the dimensions of occupational burnout as well as sociodemographic and work-related characteristics. (4) Conclusions: The process of occupational burnout varies among people in various high-risk occupations and due to sociodemographic characteristics. The internal differentiation of people representing high-risk professions requires different psychological interventions and preventive measures.

## 1. Introduction

Scientific reports on the level of stress among people employed in the European Union show that about 30% of them experience high levels of stress and are exposed to occupational burnout [1]. Currently, occupational burnout is referred to as the “epidemic of the present times” due to its widespread occurrence and negative impact on the health of the individual [2], lower efficiency at work, and employee turnover. This phenomenon is also associated with interpersonal problems between workers, a greater number of errors at work, absenteeism and consequently significant financial costs, as well as mental and physical costs of employees [3,4]. In line with the assumptions of occupational burnout regarding Job Demands–Resources (JD-R) [5], each professional environment has its own factors influencing employees’ wellbeing and level of stress. These factors form two broad categories: job demands and job resources. Sometimes work requirements that involve significant efforts from the employee cause the accumulation of a high level of occupational stress. The problem with professional requirements is that they lead to a depletion of energy needed to meet them. One of the functions of organizational resources at work is to reduce the costs incurred by the employee related to energy depletion during work. Labor resources are also used to support employees’ processes, motivate them to perform their duties, and increase their commitment to work. Even when job requirements are high, the presence of organizational resources within the work environment protects the employee from exhaustion and health problems. Burnout emerges as a prolonged response to chronic interpersonal stressors on the job [6]. This is a result of the depletion of mental and physical energy as well as the cognitive resources of a person [7].

Occupational burnout syndrome is defined as a psychosomatic state of a working person characterized by a cynical attitude towards work values as well as a lack of hope for effective work performance. The symptoms of occupational burnout develop as a consequence of workloads that increase until a person’s psychophysical resources are depleted, which decreases his or her motivation to engage with work. The negative impact of job requirements is moderated by mental, physical, social, and organizational resources [8,9]. A scarcity of resources needed to balance job requirements is conducive to employee burnout. People performing professions with extremely high requirements (responsibility for the life of another person or financial responsibility, precision of action, working under time pressure) as well as working in a position that exposes them to the chronic effects of emotional stressors are particularly at risk [10]. These professions include call takers and dispatchers, air traffic controllers, and firefighters [10,11,12].

ECDs must quickly identify specific constellations of risk indicators for human health and life through the collection of critical information and the effective delivery of appropriate first aid instructions. The health and life of another person may depend on the correct selection and evaluation of the obtained information [13,14]. A conversation with a traumatized person may give the operator symptoms of stress and emotional disorders [15,16]. Symptoms of burnout syndrome are indicated as the main cause of low retention in operator positions and high rates of sick leave [15]. Operators report lower job and life satisfaction compared to other employees (op, cit.). American research has shown that 42% of call takers and dispatchers (ECDs) assess their work as “stressful and very stressful”, 47% “demanding”, and 14% “extremely demanding”, regardless of gender and length of service [13].

Air traffic control has been classified as the fourth most stressful job [17,18]. Mental workload, fatigue, stress, situation awareness, and decision making are human factors that influence a person’s performance and result in job errors [19]. Errors in the subjective assessment of stress level and a drop in the efficiency of cognitive processes were revealed. An increased level of psychological stress impairs cognitive skills, such as attention and decision making [20]. The most important effects are health deterioration, symptoms of occupational burnout syndrome, and the occurrence of risky situations at work.

In the case of air traffic controllers, there is a possibility of “loss of picture,” which is the correct link between the idea of an aircraft’s position in the controller’s mind and its actual position. Just as many mistakes are made in situations of underload, which raises the question of the optimal stress level for cognitive function efficiency [20].

Studies have shown that firefighting is also one of the professions at a high risk of serious psychological consequences [12]. In a group of firefighters, there are often serious mental health symptoms, such as post-traumatic stress disorders (PTSD), occupational burnout syndrome, and depression. The workers encounter life-threatening situations, and must function in a state of persistent tension, constantly ready to be called out to perform rescue operations. Research [21] showed that occupational stress among firefighters and family-work conflicts were predictors of occupational burnout, which in turn was associated with a decrease in behavior conducive to work safety.

Accumulating job stress that is not balanced by personal and organizational resources may lead to the development of occupational burnout syndrome. Researchers have focused on the detection of sociodemographic determinants of burnout in many professions. In the group of ECDs, the risk factors for burnout include young age, female gender, low education level, and lack of social and family support [22]. In the group of air traffic controllers, the risk factors are young age, female gender, work-family conflict, having children, and more work experience [23,24]. In the group of firefighters, weak physical condition, young age, and less work experience were risk factors of burnout [25,26].

Most studies related to burnout syndrome focus on the analysis of variables. The relationships are analyzed between (a) the respondents obtaining results consistent with the pattern characteristic for burnout using cut-off points and (b) the variables assumed as predictors. The research is aimed at discovering the inter-individual variability among the results confirming or excluding the presence of burnout syndrome. Results that do not match the template (other than high-medium-low) are rejected from the analysis. An alternative approach is to focus on individualized employee subpopulations (person-oriented approach). The results here show the qualitatively distinct configurations of the burnout components. Half-symptomatic profiles are examined. It becomes possible to compare the so-called hidden burnout profiles in each occupational group, as well as to verify assumptions regarding the chronology of the occurrence of individual burnout symptoms. The analysis of burnout profiles has so far been carried out for a group of teachers [27,28], healthcare workers [29], nurses [30], office workers, engineers and managers [30], and psychologists [31]. The constellations of symptoms may differ in individual professions due to differences in the specificity of the work performed. Meanwhile, it is important to recognize the symptoms that appear in various dimensions of burnout early. In our research we wanted to find out about burnout profiles in a group of emotionally burdensome occupations. Purvanova i Muros [32] observed more frequent declarations of emotional exhaustion and physical exhaustion from work among women, but scientific research does not explain whether women report these signs because of work-home conflicts or other reasons. The last of these conflicts include a greater workload, an inability to meet professional expectations (restrictions in career advancement, assignment to boring and monotonous tasks), or a mismatch between the work performed and the personality traits of women. Both genders may experience burnout differently: women through signs of emotional exhaustion, men through signs of cynicism and depersonalization [33]. The results of the research indicate that, rather than the difficulty in fulfilling gender-related family responsibilities, the complexity of the social roles performed may explain the causes of occupational burnout for both women and men [34].

According to the model of occupational burnout by Maslach [35], the discussed phenomenon is a process of successively occurring negative changes in the area of mental health and behavior of workers. Our study had two goals. The aim of the research was to distinguish profiles that will reflect the types of occupational burnout among representatives of air traffic controllers, emergency number operators, and firefighters [goal 1], and to evaluate the differences between the representatives of the selected profiles in terms of selected sociodemographic variables [goal 2]. Regarding the study hypothesis, bearing in mind the information presented previously in the introduction of the article, the following research hypotheses were formulated:

**Hypothesis 1** **(H1).** *On the basis of the value of the LBQ subscales, it is possible to extract profiles that reflect the types of occupational burnout among representatives of air traffic controllers, operators of emergency numbers, and firefighters. It was assumed that individuals belonging to the identified profiles will differ in terms of the dimensions of occupational burnout: exhaustion, lack of commitment, ineffectiveness, and disappointment.*

**Hypothesis 2** **(H2).** *Individuals belonging to the selected profiles (within the analyzed professions) will differ statistically significantly in terms of sociodemographic variables (age, sex, fertility, and seniority).*

## 2. Materials and Methods

The sample of this study was composed of *n* = 1239 employees; 576 professional firefighters (100% male) aged between 20–58 years old with a mean age of M = 35.26 (SD = 6.74); 106 air traffic controllers (17.9% women, 82.1% men) aged between 24–64 years old with the mean age of M = 39.32 (SD = 8.80); 546 ECDs (57.5% women, 42.5% men) aged between 19–65 years old with the mean age of M= 34.37 (SD = 8.14). All participants were active workers. Most respondents had professional experience ranging from 1 to 5 years, were childless, and were in their first marriage (Table 1).

After signing consent to participate in the study, participants received a package of questionnaires. The responders were informed about the purpose of the study and that they would be able to withdraw from the study at any time. Respondents took an average 15 min to complete the package of questionnaires. The study design and protocols were analyzed and approved by the Ethics Committee of Jagiellonian University (decision No. 1072.6120.23.2017) and were carried out in accordance with the recommendations of the APA Ethics Code.

In the case of the group of firefighters, the study was conducted together with psychologists employed in the State Fire Service from 12 provinces of Poland during the period from January to December 2019. All participants who agreed to take part in the study completed the set of questionnaires. All sets of the questionnaires (576) were correctly filled in and sent back to the researchers. In the case of the group of air traffic controllers, questionnaires were sent to all Polish controllers working in 18 air traffic control centers of the Polish Air Navigation Services Agency (PANSA). A total of 609 sets of questionnaires were sent, of which 340 were sent back (55.88%) and 106 were filled in properly (incomplete data was rejected, the return rate was 18.1%). The data were collected between March and June 2019. The data for the group of ECDs were collected between January and May 2020 among operators working in 17 public safety answering points (PSAPs) in Poland. In total, 800 sets of research tools consisting of information about the study, standardized research questionnaires, a demographic survey, and an invitation to participate were sent. Five hundred fifty-eight sets of questionnaires were returned, of which 546 were correctly completed from 14 public safety answering points (PSAPs) (a return rate of 68.25%).

The set of questionnaires consisted of two sections. In the first demographic data (gender, age, rate of having children, marital status) and work-related data (length of service) were collected. The second section consisted of a questionnaire for the measurement of occupational burnout syndrome.

The level of burnout was assessed using the Polish version of the Link Burnout Questionnaire (LBQ) created by the Laboratory of Psychological Tests of the Polish Psychological Society [36]. The measure consists of 24 items in relation to which the subject responds on a 6 point Likert scale (1—never, 2—rarely, 3—once (or more) during a month, 4—more or less once a week, 5—several times a week, 6—every day). The questionnaire has 4 subscales according to the 4 dimensions of occupational burnout: psychophysical exhaustion (PE)—the dimension of assessing one’s own psychophysical resources, one end of which is described by exhaustion and the other by being active and full of energy; relationship deterioration (RD)—describes the quality of customer relations, at one end of this subscale we deal with the objective treatment of customers and at the other end with commitment; professional inefficacy (PI)—the dimension relating to the assessment of one’s own professional competences, one end of this dimension is characterized by a sense of effectiveness at work, and the other by a feeling of ineffectiveness; and disappointment (DI)—the dimension of existential expectations related to the specific motivation of people choosing professions related to helping others, at one end of this dimension we deal with passion and enthusiasm for work, and on the other with disappointment. Each subscale measures a range between low (6 pts) and high (36 pts) severities.

The LBQ includes four indicators: the higher the score, the greater the intensity of each dimension of burnout. The Polish version of the LBQ questionnaire has good psychometric properties. The scale of DI (Cronbach’s α = 0.84) has the highest internal reliability and the scale pertaining to the PI (Cronbach’s α = 0.68) has the lowest internal reliability. In our research, The Cronbach’s α indexes for air traffic controllers were: for the subscale of psychophysical exhaustion—0.81; for the subscale of relationship deterioration—0.67; for the subscale of professional inefficacy—0.86; for the subscale of disappointment—0.60. In the group of firefighters, the Cronbach’s α indexes were: for the subscale of psychophysical exhaustion—0.82; for the subscale of relationship deterioration—0.73; for the subscale of professional inefficacy—0.60; for the subscale of disappointment—0.8. In the case of the group of ECDs the Cronbach’s α indexes were: for the subscale of psychophysical exhaustion—0.84; for the subscale of relationship deterioration—0.68; for the subscale of disappointment—0.86; for the subscale of professional inefficacy—0.63.

A statistical analysis of the results was done using SPSS (IBM SPSS 25 Statistic, Chicago, USA). Descriptive statistics of the tested variables were used to characterize the study group. They included: mean value (M), standard deviation (SD), and group size (n). In order to determine the extent to which the distributions of the analyzed variables were close to normal, the Kolmogorov-Smirnov statistical test was used. The measures of symmetry of distributions (skewness) and the concentration of distributions of the studied variables (kurtosis) were analyzed. The central limit theorem was also invoked; distributions close to normal were considered to be those whose kurtosis and skewness ranged from −2 to 2 [37]. In order to estimate the significance of differences between the means, the F-Fisher statistic was determined based on the analysis of variance. The isolation of profiles with specific characteristics of occupational burnout was possible thanks to the k-means cluster analysis. The chi-square test of independence was also used.

## 3. Results

### 3.1. Extraction of Clusters

The first stage of the analysis was to check the distributions of the analyzed variables. For this purpose, the Kolmogorov-Smirnov test was performed (Table 2).

Referring to the central limit theorem, it was assumed that the analyzed variables had a distribution close to normal, therefore parametric tests were used in further analyses. A different than normal distribution was found for two variables in the EDs group: psychophysical exhaustion and professional inefficacy. The values of the LBQ subscales were used to distinguish profiles that reflected the types of occupational burnout among representatives of air traffic controllers, ECDs, and firefighters (H1). Nine types of occupational burnout were distinguished, three in each occupational group (Table 3 and Table 4).

**Figure 1:**
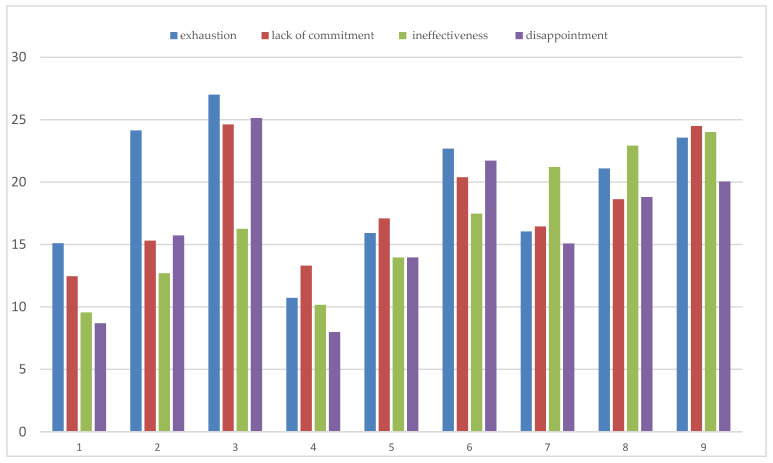
Intensification of occupational burnout dimensions in the analyzed profiles. Note. Profiles in a group of Air Traffic Controllers: 1—engaged, with a reduced sense of effectiveness, 2—reduced sense of effectiveness, 3—burned out with a low sense of effectiveness; profiles in a group of firefighters: 4—at low risk of burnout with a psychophysical exhaustion component, 5—exhausted with a moderate tendency to disappointment, 6—burned out, ineffective, and uninvolved; profiles in a group of ECD: 7—not burned out, 8—at risk of professional burnout, 9—exhausted with a tendency to disappointment.

The extracted profiles were compared with each other (H1), taking into account the dimensions of occupational burnout: exhaustion, lack of commitment, ineffectiveness, and disappointment (Table 4). The comparisons of the dimensions of occupational burnout were carried out in the profiles within the examined occupational groups. Air traffic controllers, firefighters, and ECDs included in the separate profiles differed significantly in terms of all dimensions of occupational burnout. The highest intensity of most dimensions of burnout—lack of commitment, lack of a sense of effectiveness, and disappointment—was presented by the representatives of ECDs (profiles 7–9). On the other hand, the highest psychophysical exhaustion concerned profiles 2 and 3, i.e., among representatives of air traffic controllers (Figure 1 and Table 4).

### 3.2. Diversification of Demographic Variables and Work Experience in Separate Clusters

Then, it was checked what differentiation occurs within the selected groups due to socio-demographic variables. The following factors were taken into account: age, gender, rate of having children, and work experience (H2).

#### 3.2.1. Age

In the light of the presented research results, the differences in terms of age between people representing the identified nine profiles of occupational burnout were statistically insignificant (F = 0.26; *p* = 0.772). Additionally, intra-group differences in terms of age between people representing the three separate profiles of occupational burnout within occupational groups (air traffic controllers: F = 0.12, *p* = 0.889; firefighters: F = 1.32, *p* = 0.267; ECDs: F = 2.48, *p* = 0.085) were statistically insignificant.

#### 3.2.2. Gender

The presented research results revealed that the differences in terms of gender between people representing individual burnout profiles within the three occupations were statistically insignificant. Taking into account the intra-group comparisons, statistically significant differences were observed in the group of air traffic controllers only in the first profile, with a low risk of burnout with a component of psychophysical exhaustion (χ^2^ = 574.64; *p* < 0.001). In the first profile, there was a significantly higher percentage of men than women. In the remaining profiles, these differences were statistically insignificant. In the group of firefighters in each cluster there were only men. In the group of ECDs, comparisons in terms of gender were not statistically significant within individual profiles of occupational burnout. Comparisons within the profiles turned out to be statistically significant. In each of the three profiles (profile 7 χ^2^ = 367.94; *p* < 0.001, profile 8 χ^2^ = 1693.37; *p* < 0.001, profile 9 χ^2^ = 1078.84; *p* < 0.001), there were more women than men.

#### 3.2.3. Having Children

In the light of the presented research results, the differences in the rate of having children representing specific profiles of occupational burnout within three occupations are statistically insignificant. Taking into account the differences within the profiles, in the group of air traffic controllers there were statistically significant differences only in the first profile of occupational burnout—low risk of burnout with a component of psychophysical exhaustion. In the remaining profiles, these differences were statistically insignificant. In **profile no. 1—at low risk of burnout with exhaustion component**, the greatest number of controllers had two children (Table 5). In the group of firefighters, the differences in the number of children in individual profiles of occupational burnout were statistically insignificant. Taking into account the differences within the profiles, statistically significant differences were noted within each profile of occupational burnout. In each of the profiles, the greatest number of firefighters had two children (Table 5). In the group of ECDs, the differences in the number of children in individual profiles of occupational burnout were not statistically significant. Taking into account the differences within the clusters, statistically significant ones were noted in each profile of occupational burnout. In each of the profiles, the greatest number of operators had no children (Table 5).

#### 3.2.4. Seniority

In the light of the presented research results, the differences in terms of work experience among participants representing particular profiles within the professions of air traffic control and ECD were statistically insignificant. Differences in terms of seniority between profiles in the group of firefighters were demonstrated. The firefighters representing the **profile 6—exhausted with a tendency to disappointment**—had a significantly longer period of service than the firefighters representing **profile 4—not burned out professionally**. Moreover, differences within individual clusters in each of the researched professional groups were shown. Among air traffic controllers, there were statistically significant differences in terms of seniority between those representing profile **1—at low risk of burnout with a component of psychophysical exhaustion**, and **profile 2—exhausted with a moderate tendency to disappointment**. In both profiles, the highest number of auditors had been working in the profession for 10 to 20 years (Table 5). Among firefighters, statistically significant differences were found within each profile of occupational burnout. In each profile, the length of service of its representatives ranged from 10 to 20 years (Table 6. Among the ECDs, statistically significant differences were found within each profile (H = 5.44; *p* = 0.006). In each profile, the greatest number of participants had work experience in the range of one to five years (Table 6).

## 4. Discussion

In light of the presented results, significant differences in the intensity of occupational burnout dimensions were observed in the studied professional groups: air traffic controllers, firefighters, and ECDs. Profiles of occupational burnout have been distinguished. These may reflect on the specifics of the impact of job requirements and resources on an employee and various stages of burnout. This is confirmed by the fact that different groups of individuals performing high-risk occupations are internally diverse [38]. This should be reflected in the mental support system dedicated to individuals performing various high-risk occupations and the prevention of burnout in these groups [38].

In the group of ECDs, three profiles of occupational burnout were distinguished. **Profile 1**—**engaged, with a reduced sense of effectiveness**—is characterized by an average level of commitment to relationships with clients and colleagues, a moderately high level of exhaustion, a sense of effectiveness in work activities, and an average level of disappointment.

**Profile 2**—**reduced sense of effectiveness**—is characterized by an average-high sense of ineffectiveness, an average commitment to customer relations, a sense of disappointment, and psychophysical exhaustion.

**Profile 3**—**burned out with a low sense of effectiveness**—is characterized by high levels of ineffectiveness, moderately high levels of exhaustion, and little involvement in relationships with clients and colleagues. At the same time, this profile is characterized by an average level of job disappointment. In each of the distinguished profiles, a lowered or low level of work efficiency is perceived. It can be hypothesized that ECDs are not in direct contact with their interlocutors, nor do they receive direct help, which may translate into a low sense of efficiency at work. Another reason for the low sense of efficiency in the work of operators may also be the fact that they do not have the possibility to choose their colleagues, and their impact on working conditions and wages is significantly limited. Mostly, this is due to the nature of the work performed and the strong institutionalization of the ECD profession [39,40].

The lower involvement of people belonging to profile 3 with clients and colleagues may be due to, among others, the length of contact with the person dialing the emergency number, which on average in Poland is less than two minutes [41]. In the light of the results of the studies conducted so far, the experience of chronic, high intensity stress in the group of ECDs was associated with the development of anxiety and depressive disorders in this group [42]. The lower involvement of ECDs from profile 3 with clients and colleagues, their treatment of people as objects, or their indifference towards people can be consequences of emotional exhaustion or a defense mechanism aimed at protecting their own emotional resources [43,44,45]. The statistics also show that there is a high employee turnover in the group of ECDs. Therefore, the work environment is characterized by a high degree of variability associated with employee turnover, which in turn may translate into lower support provided by colleagues [41]. ECDs also have lower social recognition. Employees’ experiences prove that they are not treated as equal partners of representatives of emergency services [41,42].

Despite numerous burdens in the professional environment, ECDs belonging to particular profiles were characterized by an average level of psychophysical exhaustion, which is described as: fatigue, a sense of tension, being under pressure [44]. This can be explained by the presence of detailed procedures for the activities performed and clear rules of work, which reduce the perceived burden caused by professional requirements.

Performing the work of an air traffic controller has been classified as the fourth most stressful profession, because many people’s lives depend on the efficiency of their cognitive processes and decisions made [46,47]. Air traffic controller errors are believed to play a significant role in 24% of air crashes. However, if we take into account aviation incidents, the share of air traffic controllers’ errors is significantly higher and can be even 82% [48,49].

In the group of air traffic controllers, three profiles of occupational burnout were distinguished. **Profile 4**—**at low risk of burnout with a psychophysical exhaustion component**—was constituted by participants who experienced the work environment as positive, did not tend to be disappointed, were involved in relationships with clients and colleagues, and felt that they were effective in the activities undertaken; however, they had a moderately severe level of psychophysical exhaustion.

**Profile 5**—**exhausted with a moderate tendency to disappointment**—consisted of participants characterized by a high sense of psychophysical exhaustion, moderate disappointment, and only minor difficulties in the areas of commitment and effectiveness.

**Profile 6**—**burned out, ineffective, and uninvolved**—was represented by participants with the highest sense of exhaustion in the group of controllers, reduced commitment to customer relations and sense of effectiveness, and an average level of job disappointment. In each of the three profiles of occupational burnout of air traffic controllers, average or high levels of psychophysical exhaustion were observed. The effectiveness of air traffic control is essentially influenced by physical fatigue and the mental state of an individual [50,51]. The decision-making process of air traffic controllers who are chronically overburdened by workplace demands that exceed their emotional resources can lead to a series of negative consequences. The most important of them are the deterioration of health, the development of the burnout syndrome, and the occurrence of risky situations at work [20]. In emergency situations, the air traffic controller has to make decisions while in contact with the pilot of the plane, which is often accompanied by strong emotions and aggressive communication. It has been proven that verbal communication based on aggression is an important risk factor for the development of occupational burnout, as is fatigue and overwork [50,52]. In addition, the work of an air traffic controller is shift work and requires work at night. Research carried out on a group of Polish air traffic controllers has shown that they are a group at risk of sleep disorders [53]. Research conducted in the Netherlands in a group of shift workers has shown that they report a 50% higher level of fatigue and a deterioration of sleep quality by 90% compared to non-shift workers [54,55]. It seems, therefore, that the specificity of the work of an air traffic controller—burdened with a high level of responsibility, experiencing strong emotions and a large number of stressors, and working in a shift system—may contribute to a greater intensity of psychophysical exhaustion.

Research conducted in Poland among firefighters showed that they displayed a lower level of occupational burnout in all its dimensions compared to employees of other social services [12,25]. In addition, links between emotional exhaustion and depersonalization among firefighters and the experience of stressors, such as workload, lack of rewards, insecurity caused by work organization, social contacts, sense of threat, and responsibility, have been demonstrated [25].

Firefighters constituted internally diverse profiles of occupational burnout. In this group, three profiles of occupational burnout were also distinguished. **Profile 7**—**not burned out**—was represented by people involved in relations with clients and colleagues, experiencing a sense of the effectiveness within interactions at work and job satisfaction, and with a lower level of psychophysical exhaustion. **Profile 8**—**at risk of professional burnout**—was characterized by firefighters who showed symptoms of psychophysical exhaustion, experienced moderate difficulties in engaging in relationships with clients and colleagues, and had a moderate disenchantment with work. They felt average efficiency at work.

**Profile 9**—**exhausted with a tendency to disappointment**—was represented by individuals who experienced increased psychophysical exhaustion associated with work and a moderately low level of effectiveness and commitment to customer relationships. Firefighters in this profile had a moderately high level of disappointment.

In firefighters’ burnout profiles 5 and 6, there were moderate levels of disappointment with work and a large reduction in commitments to relationships with clients and colleagues. Commitment can be read as an indicator of job fulfillment, while burnout suggests difficulties, failures, and lack of success at work [29]. It seems that high job disappointment may be related to a relatively low salary, which is often associated with the need to combine several different jobs. The results of the studies conducted so far indicated that a higher level of commitment was a significant factor protecting against the development of occupational burnout and its dimensions, such as exhaustion and a sense of depersonalization [56].

It can be hypothesized that the constellation of the intensity of occupational burnout dimensions of firefighters representing profile 4 is characteristic of a mentally resistant personality—highly open-minded, capable of involvement, and having a sense of agency, which is a resource against the burnout experience [52]. The results of research by Polish firefighters [57] proved that they constitute a professional group convinced of their own competences and, therefore, they perceive stressful situations as within their control. This fact was confirmed by the results of the studies which showed that the surveyed firefighters in individual burnout profiles were accompanied by a moderate or moderately low level of conviction about their effectiveness at work.

The profiles identified in the group of firefighters reflect the subsequent stages of burnout. Burnout is processual in nature [35,45]. It has been noted that the first stage of burnout is exhaustion, which in turn leads to lower involvement, depersonalization, and cynicism. At the same time, but partially independently, satisfaction with professional achievements decreases, which shapes the belief that the actions taken are not very effective [58]. It seems that the course of occupational burnout is diversified in the analyzed professional groups. A characteristic feature of firefighters exhibiting the features of burnout was a strong sense of disappointment in the studied sample. The profiles unfired, at risk of burnout, and burned out with a tendency to disappointment illustrate the successive stages of burnout in this group. On the other hand, a distinctive feature of burnout profiles in the group of ECDs was a reduced sense of effectiveness. It can also be concluded that the process of burnout is in line with Maslach’s theory in the case of ECDs—a high sense of exhaustion translates into a lack of commitment and depersonalization. Perhaps the tendency to lower involvement is a mechanism that protects against experiencing excessively strong affective states that may significantly interfere with the course of cognitive processes and effective communication. It seems that this requires further in-depth analyses.

The obtained research results allow for the acceptance of hypothesis 1, in which we assumed that on the basis of the value of the LBQ subscales, it is possible to distinguish profiles that reflect the types of occupational burnout among representatives of air traffic controllers, emergency number operators, and firefighters, and that people belonging to the selected profiles will differ with respect to the dimensions of occupational burnout. The development of occupational burnout is influenced not only by factors related to the profession, but also by socio-demographic variables [35,36]. The distinguished profiles of occupational burnout were not differentiated by age. The results of research on the importance of age for occupational burnout obtained so far are inconclusive. Some of the research results showed that age is most strongly associated with occupational burnout among the socio-demographic variables [35,36]. It has been proven that younger workers are at higher risk of burnout [58,59]. This may be related to the confrontation of idealistic expectations towards work and reality as well as a lack of work experience. Thus, the older group included participants who were able to develop appropriate adaptation mechanisms [45,58,59,60]. The results of other studies conducted in the group of nurses did not confirm that the demographic data were significantly related to occupational burnout [59].

According to the research results of Stanisława Tucholska [58], burnout syndrome is more concerned with women than men. More detailed studies showed that women scored higher on emotional exhaustion and men scored higher on depersonalization [3,44,59]. On the other hand, the results of the research by Lenzo et al. [61] showed that gender does not differ in regards to the dimensions of occupational burnout. Additionally, in our own research, it was shown that gender did not differ in regards to the severity of occupational burnout within the profiles described.

Marcionetti et al. [62] showed that people with children perceived workload to be higher, but they were also accompanied by greater optimism and satisfaction with life. In the light of the research results obtained within the framework of the selected clusters, only a slight differentiation in terms of having children was noted. In the low-risk profile of burnout with a component of psychophysical exhaustion, the greatest number of air traffic controllers had two children. Additionally, in the group of firefighters, each profile was represented most frequently by people with two children. In turn, in the group of ECDs, the largest number of respondents was childless.

It is also indicated that seniority is an important factor related to professional burnout. Firefighters representing the exhausted profile with a tendency to be disappointed had a significantly longer length of service than the firefighters representing the unfired profile. The research results indicate that emotional exhaustion as one of the dimensions of occupational burnout is negatively related to seniority [63].

The obtained research results allow us to partially accept the hypothesis 2, in which we assumed that people belonging to the selected profiles (within the analyzed professions) will differ statistically significantly in terms of sex, number of children, and seniority. Only the assumption that individual profiles of occupational burnout will be differentiated by age was not confirmed.

## 5. Limitation of the Study

The authors of the study are aware of their limitations, mainly regarding the methodology used. It was only a cross-sectional study with low accuracy and unable to establish cause-and-effect relationships. The fundamental limitation of the research resulted from the applied procedure of selecting the subjects. The studied groups were not equal in number and their selection was not randomized. Proportional selection was not used in the case of demographic variables. Purvanova and Muros’ [32] meta-analysis revealed gender differences in burnout that might have been harder to detect here given our reliance on male-dominated samples. The group of air traffic controllers differed from the rest in terms of numbers, which may have an impact on the generalization of the results. Self-report tools were used in the research, which is burdened with the risk of measurement errors (self-reported biases, social desirability). The study did not investigate the effects of social and economic events that could have influenced their outcomes. Cross-sectional surveys based on a questionnaire have a risk of bias due to only one source of data, the longitudinal research would make it possible to test whether membership in profiles remains stable over time. Other individualized employee subpopulations-oriented studies are needed to confirm the generalizability of the profiles.

## 6. Conclusions

Through the conducted research, we have tried to show that the process of occupational burnout is diversified in terms of occupation and socio-demographic data. It seems that specific professions require the diversification of psychological interventions. The same diversifications are needed in terms of preventive therapies undertaken and their adaptation to given professional groups. It also seems that the ECDs are the group particularly exposed to professional burnout.

## Figures and Tables

**Table 1:** Characteristics of the group of participants N = 1239.

Variables	Air Traffic Controllers		Firefignters		ECDs		Total	
**Demographic Data**								
**age *M*(*SD*)**	39.47	8.79	35.25	6.73	34.40	8.11	36.32	7.89
**work experience** **interval**	*n*		*n*		*n*		*n*	
**1–5 years**	22		45		362		429	
**6–10 years**	24		152		184		360	
**11–20 years**	61		238		12		311	
**>20 years**	-		130		-		130	
**having children** **category**	*n*		*n*		*n*		*n*	
**0**	30		169		169		368	
**1**	25		139		95		259	
**2**	38		226		78		342	
**3**	11		39		14		64	
**4**	3		6		2		11	
**5**	-		-		1		1	
**martial status** **category**	*n*		*n*		*n*		*n*	
**single**	14		-		166		180	
**cohabiting**	16		-		146		162	
**first Married**	64		-		227		291	
**second and next Married**	13		-		12		25	
**widowed**	-		-		3		3	
**LBQ factors**	*M*	*SD*	*M*	*SD*	*M*	*SD*	*M*	*SD*
**PE**	17.95	6.03	15.70	5.60	20.82	3.99	18.18	5.55
**RD**	13.98	5.19	16.54	4.39	20.04	4.31	17.89	4.88
**PI**	10.78	4.06	13.45	4.39	22.91	3.39	17.46	6.33
**DI**	11.43	5.78	13.70	6.12	18.42	3.44	15.62	5.69

PE-psychophysical exhaustion; RD-relationship deterioration; PI-professional inefficacy; DI-disappointment.

**Table 2:** Normality test for dimensions of occupational burnout.

Variables	*K-S*	*p*	*S*	*K*
	Total			
PE	0.071	<0.01	−0.07	−0.29
RD	0.068	<0.01	−0.07	−0.10
PI	0.122	<0.01	−0.10	−0.95
DI	0.090	<0.01	−0.03	−0.47
Air Traffic Controlers				
PE	0.121	<0.01	0.61	0.25
RD	0.115	<0.01	1.00	1.31
PI	0.161	<0.01	1.32	1.70
DI	0.173	<0.01	1.35	1.52
Firefighters				
PE	0.091	<0.01	0.59	−0.07
RD	0.075	<0.01	0.10	−0.33
PI	0.076	<0.01	0.46	0.30
DI	0.100	<0.01	0.68	−0.06
		EDs		
PE	0.093	<0.01	−0.60	2.79
RD	0.072	<0.01	−0.35	1.32
PI	0.129	<0.01	−0.90	7.71
DI	0.095	<0.01	−0.54	2.55

*K-S*—the test value was carried out by Kolmogorov-Smirnov with the Lilliefors correction; *p*—statistical significance; *S*—skewness; *K*—kurtosis; PE—Psychophysical exhaustion; RD—Relation deterioration; PI—Professional inefficacy; DI—Disappointment.

**Table 3:** Euclidean distancesof distinguished clusters.

	Cluster 1	Cluster 2	Cluster 3
cluster 1	-	37.29	151.07
cluster 2	6.10	-	48.52
cluster 3	12.29	6.99	-
	cluster 4	cluster 5	cluster 6
cluster 4	-	32.30	108.65
cluster 5	5.68	-	22.77
cluster 6	10.42	4.77	-
	cluster 7	cluster 8	cluster 8
cluster 7	-	38.35	10.70
cluster 8	6.19	-	11.77
cluster 9	3.27	3.43	-

**Table 4:** Characteristics of the profiles identified in the groups of representatives of high-risk professions.

**Air Traffic** **Controllers** **(*n* = 106)**	**Profile 1—Engaged, with a Reduced Sense of Effectiveness** **(*n* = 76)**		**Profile 2—Reduced Sense of Effectiveness** **(*n* = 23)**		**Profile 3—Burned out with a Low Sense of Effectiveness** **(*n* = 8)**		***F***	**Post-Hoc**
	***M***	***SD***	***M***	***SD***	***M***	***SD***		**Tukey’s Test for Different N**
PE	15.09	3.91	24.13	4.08	27.00	5.12	65.78 ***	1 < 2, 1 < 3
RD	12.46	4.02	15.30	3.82	24.62	5.25	33.64 ***	1 < 3, 2 < 3
PI	9.56	2.82	12.69	4.29	16.25	6.47	17.25 ***	1 < 2, 1 < 3
DI	8.69	2.70	15.73	4.09	25.12	4.42	123.16 ***	1 < 2, 1 < 3, 2 < 3,
**Firefighters** **(*n* = 576)**	**Profile 4—At low risk of burnout with a psychophysical exhaustion component** **(*n* = 140)**		**Profile 5—Exhausted with a moderate tendency to disappointment** **(*n* = 233)**		**Profile 6—Burned out, ineffective, and uninvolved** **(*n* = 207)**		***F***	**Post-hoc**
	***M***	***SD***	***M***	***SD***	***M***	***SD***		**Tukey’s test for different N**
PE	10.73	2.68	15.91	3.80	22.68	4.19	548.87 ***	4 < 5, 4 < 6, 5 < 6
RD	13.31	3.81	17.09	3.18	20.39	3.78	179.94 ***	3 > 4, 3 > 5 4 < 5, 4 < 6
PI	10.17	3.03	13.96	3.33	17.47	3.94	197.97 ***	4 < 5, 4 < 6 5 < 6
DI	7.98	2.16	13.96	3.24	21.71	4.27	764.25 ***	4 < 5, 4 < 6 5 < 6
**ECDs** **(*n =* 557)**	**Profile 7—Not burned out** **(*n* = 177)**		**Profile 8—At risk of professional burnout** **(*n* = 116)**		**Profile 9—Exhausted with a tendency to disappointment** **(*n* = 265)**		***F***	**Post-hoc**
	***M***	***SD***	***M***	***SD***	***M***	***SD***		**Tukey’s test for different N**
PE	16.05	3.55	21.09	2.49	23.55	3.22	224.02 ***	7 < 8, 7 < 9, 8 < 9
RD	16.44	4.28	18.62	2.49	24.48	2.51	313.74 ***	7 < 8, 7 < 9, 8 < 9
PI	21.20	4.30	22.92	2.97	24.01	2.71	26.61 ***	7 < 8, 7 < 9, 8 < 9
DI	15.08	3.53	18.80	2.69	20.05	3.01	103.81 ***	7 < 8, 7 < 9, 8 < 9

*** statistical significance.

**Table 5:** Differences in number of children due to the profile of occupational burnout in the group of air traffic controllers, firefighters, and ECDs.

Having Children	No Children	1	2	3	4	5	General
**air traffic controllers H = 1.88; *p* = 0.390**							
**profile 1 χ^2^ = 101.44; *p* < 0.001**	19	18	29	7	3	-	76
**%**	25.00	23.68	38.16	9.21	3.95	-	100
**profile 2 χ*2* = 1.75; *p* = 0.186**	10	4	6	3	-	-	23
**%**	43.48	17.39	26.09	13.04	-	-	100
**profile 3 χ^2^ n.i.**	1	3	3	1	-	-	8
**%**	12.50	37.50	37.50	12.50	-	-	100
**firefighters H = 2.88; *p* = 0.238**							
**profile 4 χ^2^ = 268.60; *p <* 0.001**	69	45	71	20	1	-	206
**%**	33.33	21.74	34.30	9.66	0.48	-	100
**profile 5 χ^2^ = 404.61; *p* < 0.001**	65	63	94	9	2	-	233
**%**	27.90	27.04	40.34	3.86	0.86	-	100
**profile 6 χ^2^ = 281.32; *p* < 0.001**	35	31	61	10	3	-	140
**%**	25.00	22.14	43.57	7.14	2.14	-	100
**ECDs H = 2.18; *p* = 0.336**							
**profile 7 χ^2^ = 97.08; *p* < 0.001**	41	15	15	3	-	-	74
**%**	35.34	12.93	12.93	2.59	-	-	100
**profile 8 χ^2^ = 137.88; *p* < 0.001**	75	41	39	8	2	1	166
**%**	28.30	15.47	14.72	3.02	0.75	0.38	100
**profile 9 χ^2^ = 20.03; *p* = 0.001**	53	39	24	3	-	-	119
**%**	29.94	22.03	13.56	1.69	-	-	100

profile 1—low risk of burnout with a component of psychophysical exhaustion (*n* = 76); profile 2—exhausted with a moderate tendency towards disappointment (*n* = 23); profile 3—burned out, ineffective, and uninvolved (*n* = 8); profile 4—not burned out (*n* = 207); profile 5—at risk of professional burnout (*n* = 233); profile 6—exhausted with a tendency towards disappointment (*n* = 140); profile 7—committed, with a lowered sense of effectiveness (*n* = 116); profile 8—with a reduced sense of effectiveness (*n* = 265); profile 9—burned out with a high sense of ineffectiveness (*n* = 177).

**Table 6:** Differences in terms of seniority according to the profile of occupational burnout in the groups of air traffic controllers, firefighters, and ECDs.

Seniority	1–5 Years	6–10 Years	10–20 Years	More Than 20 Years	General
**air traffic controllers, H = 0.55; *p* = 0.761**					
**type 1 χ^2^ = 279.11; *p* < 0.001**	15	17	44	-	76
**%**	19.74	22.37	57.89	-	100
**type 2 χ^2^ = 8.87; *p* = 0.003**	6	5	12	-	23
**%**	26.09	21.74	52.17	-	100
**type 3 χ^2^ = n.i.**	1	2	5	-	8
**%**	12.50	25.00	62.50	-	100
**firefighters, H = 10.57; *p* = 0.005**					
**type 4 χ^2^ = 1298.47; *p* < 0.001**	32	57	69	48	206
**%**	15.46	27.54	33.33	23.19	100
**type 5 χ^2^ = 1932.45; *p* < 0.001**	17	68	101	44	230
**%**	7.30	29.8	43.35	18.88	100
**type 6 χ^2^ = 790.70; *p* < 0.001**	7	27	68	38	140
**%**	5.00	19.29	48.57	27.14	100
**ECDs, H = 5.44; *p* = 0.006**					
**type 7 χ^2^ = 1721.40; *p* < 0.001**	69	42	5	-	116
**%**	59.42	36.21	4.31	-	100
**type 8 χ^2^ = 2526.25; *p* < 0.001**	167	93	5	-	265
**%**	63.02	35.09	1.89	-	100
**type 9 χ^2^ = 817.53; *p* < 0.001**	126	49	2	-	180
**%**	71.19	27.68	1.13	-	100

## Data Availability

Not applicable.
